# Evidence-based translation in postpartum pelvic floor rehabilitation nursing: recent advances and practical integration

**DOI:** 10.3389/fpubh.2026.1818371

**Published:** 2026-06-17

**Authors:** Yufei Yuan, Huimin Su, Quanyi Long, Yuanyuan Zou

**Affiliations:** 1 Department of Obstetrics and Gynecology, The Second Hospital of Kunming, Kunming, China; 2 Department of Internal Medicine, Quanzhou First Hospital, Quanzhou, Fujian, China; 3 Faculty of Nursing, Kunming Medical University, Kunming, China; 4 Department of Neurosciences, Mental Health and Sensory Organs—NESMOS, Sapienza University of Rome, Rome, Italy

**Keywords:** adherence, continuity of care, digital health, evidence-based nursing, pelvic floor muscle training, postpartum pelvic floor rehabilitation

## Abstract

**Background:**

Postpartum pelvic floor dysfunction (PFD) is a common condition and can undermine women's physical comfort, mental health, and social functioning. As service needs grow, evidence-based translational nursing has been proposed to strengthen postpartum pelvic floor rehabilitation. This review summarizes the current evidence and compares it with routine care.

**Materials and methods:**

Recent studies and clinical practice guidelines were examined. Core applications included standardized assessment, pelvic floor muscle training (PFMT), biofeedback/electrical stimulation when indicated, psychological support, health education, and internet-enabled follow-up for continuity of care.

**Results:**

Compared with conventional nursing, evidence-based approaches appear to be associated with greater gains in pelvic floor muscle (PFM) strength and lower rates of urinary incontinence and pelvic organ prolapse (POP) in the included literature, with reduced risk of recurrence. Many reports also described improvements in anxiety/depressive symptoms, sexual function, quality of life, satisfaction, and adherence to rehabilitation programmes.

**Conclusion:**

By aligning research evidence with clinical judgment and women's preferences, evidence-based translational nursing can deliver more targeted and continuous rehabilitation and support more complete postpartum recovery.

## 1 Introduction

**Figure 1:**
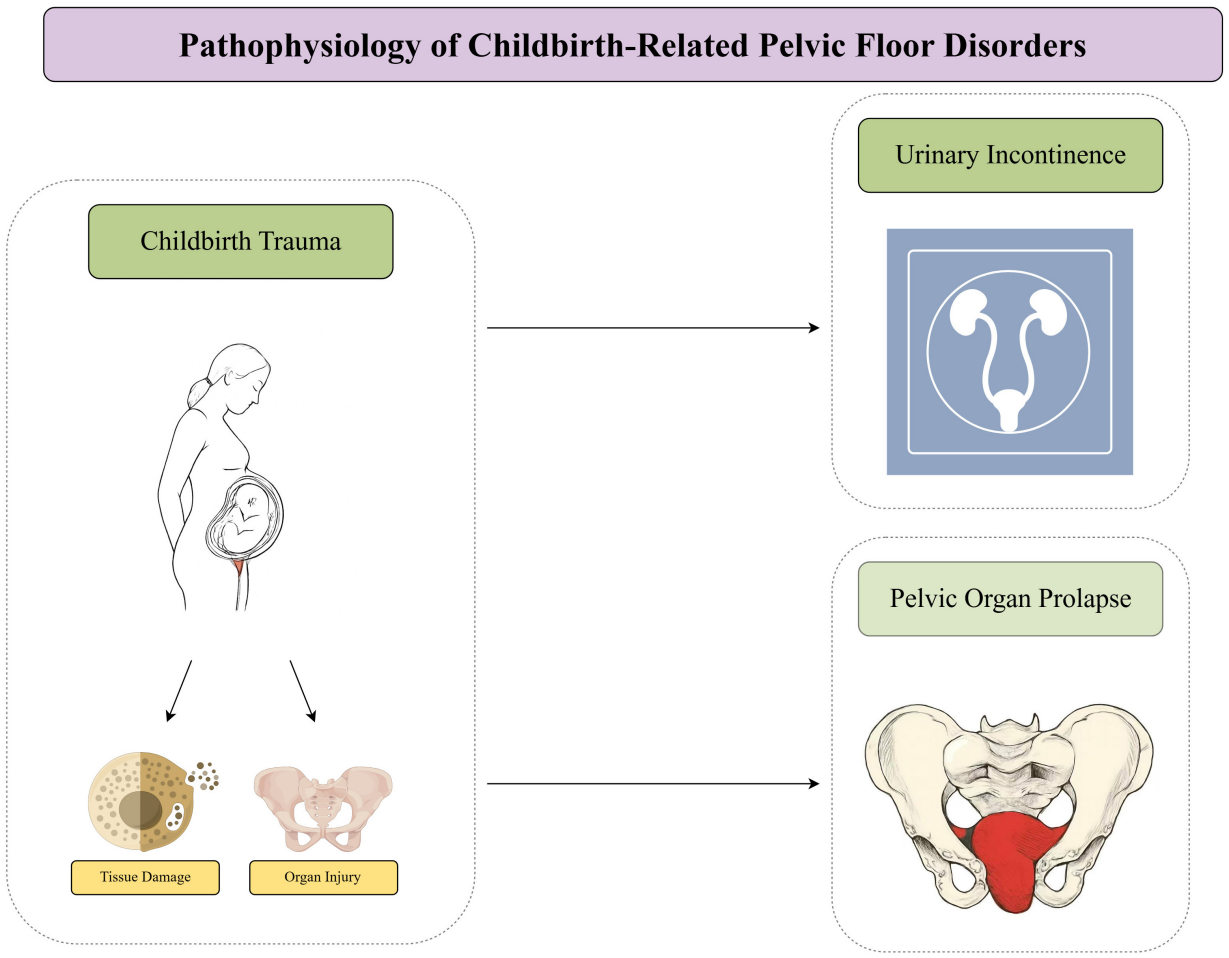
Pathophysiology of childbirth-related pelvic floor disorders. PFD, pelvic floor dysfunction; SUI, stress urinary incontinence; POP, pelvic organ prolapse.

Pregnancy and childbirth represent transformative periods in a woman's life, yet they frequently impose considerable strain on pelvic floor structures. During delivery, the pelvic floor muscles (PFM), ligaments, and fascia undergo substantial stretching, compression, or tearing, which may lead to postpartum pelvic floor dysfunction (PFD; Figure 1). PFD encompasses a spectrum of clinical manifestations, including stress urinary incontinence (SUI), pelvic organ prolapse (POP), sexual dysfunction, and postpartum urinary retention. These conditions negatively affect women's physical health, psychological well-being, daily functioning, and family life (1, 2). Early identification of high-risk factors and timely rehabilitation are therefore essential in mitigating long-term complications (3). Epidemiological data suggest that approximately one-third of women experience urinary incontinence after childbirth, and up to 10% encounter fecal incontinence (4), underscoring the importance of effective postpartum pelvic floor rehabilitation to promote holistic recovery and enhance quality of life.

Traditional postpartum care often suffers from variability and lack of standardization, which may hinder optimal rehabilitation outcomes (5). With the evolution of healthcare paradigms, evidence-based nursing (EBN) has emerged as a practice model that integrates the best available research evidence with clinical expertise and patient preferences to inform individualized care decisions (6). The introduction of EBN addresses the limitations of traditional nursing practices by systematically searching, appraising, and synthesizing scientific evidence to guide postpartum pelvic floor rehabilitation. For example, Chowdhury and Hossain (7) developed an evidence-based care pathway for postpartum pelvic floor rehabilitation, demonstrating the translational value of EBN in clinical practice.

This review examines recent advances in the application of evidence-based translation in postpartum pelvic floor rehabilitation nursing. It outlines the developmental trajectory and core principles of EBN, summarizes practical strategies in clinical settings, compares the effectiveness of evidence-based and routine nursing approaches, and highlights key directions for future research to support theoretical development and clinical implementation.

Postpartum pelvic floor rehabilitation should also be understood within the broader public health context of postpartum physical activity, sedentary behavior reduction, and health promotion, as pelvic floor exercises are commonly recommended in postpartum physical activity guidelines together with general aerobic and strengthening activities (8).

In this review, evidence-based translation refers to the process of converting research findings, clinical guidelines, clinical expertise, and women's preferences into standardized yet individualized postpartum pelvic floor rehabilitation pathways. This process involves evidence retrieval and appraisal, clinical adaptation, patient-centered education, supervised or remote implementation, repeated outcome evaluation, and continuous quality improvement. In this sense, “translation” emphasizes not only the identification of evidence, but also its practical integration into nursing assessment, intervention delivery, follow-up, and rehabilitation optimization.

### 1.1 Review design and methods

This article was designed as a narrative review of evidence-based nursing strategies for postpartum pelvic floor rehabilitation. Relevant studies, clinical guidelines, and reviews were searched in PubMed, Web of Science, Scopus, CNKI, and Google Scholar using terms related to postpartum pelvic floor dysfunction, pelvic floor rehabilitation, pelvic floor muscle training (PFMT), biofeedback, electrical stimulation, evidence-based nursing, continuity of care, and quality of life. Literature was included if it focused on postpartum pelvic floor dysfunction, rehabilitation interventions, nursing care, or related clinical outcomes. The evidence was synthesized narratively according to intervention type, outcome domain, and clinical application. As this was not a systematic review, formal risk-of-bias assessment and evidence certainty grading were not performed.

## 2 Development of evidence-based nursing

Evidence-based nursing originated from the evidence-based medicine movement that emerged in the late 1980s and early 1990s. Pioneered by David Sackett and colleagues at McMaster University, the movement emphasized the use of high-quality scientific evidence to guide clinical decision-making (9), fundamentally shifting the traditional reliance on expert opinion.

As evidence-based medicine matured and its benefits became widely recognized, nursing—being a highly practice-oriented discipline—likewise required rigorous empirical foundations to ensure the safety and effectiveness of clinical care, giving rise to evidence-based nursing. The core principles of EBN encompass four main components: (1) evidence acquisition and appraisal, involving systematic retrieval and grading of scientific literature; (2) integration of clinical expertise, ensuring evidence is applied appropriately within the nursing context; (3) patient-centered decision-making, tailoring interventions to individual needs and values (10); and (4) continuous evaluation and optimization, monitoring outcomes and adjusting care accordingly. Research further indicates that the management of PFD requires multidisciplinary collaboration—incorporating expertise from gynecology, nursing, physiotherapy, and health education—to achieve comprehensive assessment and intervention (11). Enaux et al. (12) also emphasized the importance of advanced practice nurses (APNs) within multidisciplinary teams, demonstrating their role in implementing evidence-based pelvic floor care.

## 3 Evidence-based nursing in postpartum pelvic floor rehabilitation

### 3.1 Evidence-based assessment and diagnosis

A systematic and comprehensive assessment forms the foundation of evidence-based postpartum pelvic floor rehabilitation. Nursing staff should collect detailed obstetric histories, including mode of delivery, parity, fetal size, and intrapartum complications—factors known to influence pelvic floor integrity. Handa et al. identified episiotomy, operative vaginal delivery, and perineal lacerations as independent risk factors for PFD after childbirth (13). Additional studies have reported associations between maternal age, vaginal delivery, multiparity, and the onset of SUI in the postpartum period (14–16). In addition to obstetric factors, maternal age and relevant comorbidities should also be considered during risk stratification for postpartum PFD, as these factors may influence symptom severity, rehabilitation tolerance, and recovery outcomes.

Multiple assessment tools are utilized in clinical research and practice (as shown in Table 1). Tosun et al. (17) evaluated pelvic floor muscle function using the Oxford scale to compare outcomes across nursing interventions, while Xu et al. (10) incorporated surface electromyography and functional near-infrared spectroscopy to provide objective physiological measurements. The Pelvic Organ Prolapse Quantification (POP-Q) system is widely applied to assess the degree of POP and evaluate treatment effects (18, 19). The International Consultation on Incontinence Questionnaire-Short Form (ICIQ-SF) is a validated tool for assessing urinary incontinence severity and its impact on quality of life and is frequently used in pelvic floor rehabilitation studies (20, 21). Some researchers also employ the ICIQ-Q-LF for further evaluation of incontinence (22). Sexual function is commonly assessed using the Female Sexual Function Index (FSFI), covering dimensions such as desire, arousal, lubrication, orgasm, pain, and sexual satisfaction (23, 24). Psychological evaluation tools—including the Symptom Checklist-90 (SCL-90), Self-Rating Anxiety Scale (SAS), and Self-Rating Depression Scale (SDS)—have been adopted by Kang (25) and Xie et al. (26) to examine emotional well-being and the influence of nursing interventions on anxiety and depressive symptoms among postpartum women.

**Table 1:** Comparison of common assessment tools.

Assessment tool	Purpose	Advantages	Disadvantages	Target population
Oxford score	Assessing pelvic floor muscle function	Simple and widely used	Only reflects muscle strength, not the full pelvic floor function	Postpartum women, especially those with pelvic floor dysfunction
ICIQ-SF questionnaire	Assessing urinary incontinence symptoms and their impact on quality of life	Provides a comprehensive assessment of urinary incontinence and its impact on daily life	Limited in assessing the full impact of urinary incontinence	Postpartum women with urinary incontinence
FS-FI scale	Assessing sexual function (e.g., libido, orgasm)	Comprehensive assessment of sexual quality, including multiple dimensions	Subjective and may be influenced by individual differences	Postpartum women with sexual health concerns
SCL-90	Assessing maternal psychological state (e.g., anxiety, depression)	Standardized scale, widely used in psychological assessment	Does not solely reflect the maternal mental health	Postpartum women requiring psychological assessment
SAS	Assessing maternal anxiety	Specifically evaluates anxiety, quick diagnosis	Focuses only on anxiety, lacks evaluation of other emotional states	Postpartum women with symptoms of anxiety

### 3.2 Selection and implementation of evidence-based interventions

#### 3.2.1 Pelvic floor muscle training (PFMT)

Pelvic floor muscle training—particularly Kegel exercises—constitutes the foundational and most widely endorsed intervention in postpartum pelvic floor rehabilitation. Through repeated voluntary contraction and relaxation of the pelvic floor muscles, PFMT enhances muscle strength, endurance, and neuromuscular coordination. Numerous studies have confirmed its clinical effectiveness. Evidence indicates that PFMT reduces postpartum urinary retention, alleviates perineal discomfort, and improves patient satisfaction, while its non-invasive, equipment-free, and easy-to-learn nature facilitates broad clinical adoption (27–29). Studies have also indicated that postpartum pelvic floor muscle training significantly enhances pelvic floor muscle contractility, improves sexual function, and reduces the incidence of postpartum stress urinary incontinence and associated depressive symptoms. Despite heterogeneity across clinical trials, PFMT has demonstrated positive effects in preventing pelvic floor dysfunction—particularly urinary incontinence—during both the prenatal and postnatal periods (30, 31). However, although many postpartum women possess adequate knowledge and generally positive attitudes toward PFMT, household responsibilities and childcare demands often hinder regular practice. This highlights the need to consider real-world adherence challenges when promoting PFMT in clinical settings (32).

#### 3.2.2 Biofeedback and electrical stimulation therapy

Biofeedback and electrical stimulation are important adjunctive modalities that significantly enhance the effectiveness of pelvic floor muscle training. Biofeedback converts real-time pelvic floor muscle activity into visual or auditory signals, enabling postpartum women to accurately perceive and control muscle contraction and relaxation, thereby correcting improper training techniques. Electrical stimulation applies low-frequency currents to activate pelvic floor nerves and muscles, promoting neuromuscular recovery, enhancing muscle strength, improving local circulation, and relieving pain. To avoid terminological ambiguity, biofeedback, electrical stimulation, and vaginal cone training are described in this review as device-supported adjunctive rehabilitation modalities, whereas active PFMT refers to voluntary pelvic floor muscle contraction-based training.

Evidence shows that combining biofeedback and electrical stimulation with vaginal cones markedly improves pelvic floor muscle strength and sexual function in women with postpartum pelvic floor dysfunction, and effectively reduces the incidence of urinary incontinence (33, 34). Other studies have demonstrated that incorporating low-frequency electrical stimulation and biofeedback into routine pelvic floor rehabilitation significantly enhances muscle strength and reduces the occurrence of pelvic organ prolapse and urinary incontinence (35). Romeikiene and Bartkevičiene (30) similarly reported that postpartum pelvic floor rehabilitation effectively decreases prolapse and incontinence in women with pelvic floor dysfunction.

Early pelvic floor muscle training combined with electrical stimulation has been shown to prevent pelvic floor dysfunction in high-risk postpartum women, indicating a synergistic therapeutic effect (36). In addition, integrating rehabilitative exercises with low-pressure, low-frequency vaginal electrical stimulation (DES) facilitates recovery of pelvic floor neuroelectrophysiology and tissue function. Studies using biofeedback stimulation devices alongside structured nursing interventions also report improvements in pelvic floor function and muscle strength (37). Furthermore, emerging evidence suggests that electromagnetic therapy leads to superior anatomical improvements compared with single-modality treatments (10), highlighting the growing potential of physiotherapy-based approaches.

#### 3.2.3 Vaginal cones training

Vaginal cones (or vaginal dumbbells) are commonly used as adjunct tools to facilitate resistance-based pelvic floor training. By providing graded weights, they help women engage in progressive load-bearing exercises. When combined with biofeedback and electrical stimulation therapy, vaginal cones have been shown to significantly enhance pelvic floor muscle strength and improve sexual function (38). The use of Kegel cones has also been described as an effective form of gravity-assisted feedback training, helping women develop better awareness and control of pelvic floor muscles (39).

#### 3.2.4 Psychological support and health education

Postpartum pelvic floor dysfunction is often accompanied by psychological distress, including anxiety and depression. Evidence-based nursing addresses these concerns by incorporating comprehensive psychological support and structured health education to improve women's emotional well-being. Psychological interventions are commonly integrated into postpartum pelvic floor rehabilitation, and incorporating such interventions alongside behavioral theoretical models has been shown to significantly enhance rehabilitation outcomes (40). The Information–Motivation–Behavioral Skills (IMB) model has demonstrated multiple benefits, including improved pelvic floor muscle strength, reduced pelvic organ prolapse, and enhanced sexual function. Supportive psychological interventions combined with rehabilitation programmes can also alleviate urinary retention symptoms, reduce residual urine volume, and improve both pelvic floor function and emotional status (41). The Neuman Systems Model further emphasizes the reduction of anxiety and depressive symptoms as a means to improve overall quality of life (42).

Health education likewise plays a critical role in shaping women's understanding and engagement in rehabilitation. Targeted postpartum pelvic floor health education has been associated with increased adherence to rehabilitation exercises (43). Qualitative research by Xu et al. (44) has identified key facilitators and barriers to pelvic floor rehabilitation behaviors during pregnancy, indicating that personal knowledge and prior experience are significant determinants. Although many postpartum women express positive attitudes toward pelvic floor exercise, practical obstacles remain, highlighting the need for more in-depth research on health education strategies (32). In this context, postpartum pelvic floor rehabilitation may also be linked with broader postpartum physical activity behavior, as structured education, mood support, and improved self-efficacy can help women understand the value of regular exercise, reduce psychological barriers, and maintain rehabilitation adherence.

#### 3.2.5 Internet-based continuity of care

With advancements in digital health technologies, internet-based continuity of care has emerged as an innovative approach for postpartum pelvic floor rehabilitation. A prospective cohort study examining online extended-care programmes for women with postpartum stress urinary incontinence found that participants receiving consistent online interaction demonstrated significantly greater improvements in pelvic floor muscle strength, adherence, and satisfaction compared with those receiving minimal interaction (45). This model is particularly suitable for postpartum women who require long-term, consistent rehabilitation support, as it overcomes time and geographic constraints and facilitates ongoing professional guidance, monitoring, and behavioral reinforcement.

More broadly, digital and home-based rehabilitation models may be particularly suitable for postpartum women because they reduce time, childcare, and geographic barriers while allowing repeated reminders, feedback, and remote professional support. Evidence from digital and home-based physical training research supports the broader feasibility of technology-assisted exercise delivery, although such evidence should be interpreted cautiously because it is not specific to postpartum pelvic floor rehabilitation (46). Future digital rehabilitation programmes should therefore pay greater attention to adherence monitoring, intervention quality, individualized feedback, and safety during home-based practice.

Although the reviewed interventions showed positive effects, the reporting of intervention dosage was heterogeneous across studies. Frequency, intensity, and duration were not consistently described using the same criteria, which limits direct comparison among different rehabilitation programmes. In clinical application, dosage decisions for pelvic floor muscle training, biofeedback, electrical stimulation, vaginal cone training, psychological support, health education, and internet-based follow-up should therefore be individualized according to baseline pelvic floor muscle strength, symptom severity, postpartum recovery stage, patient tolerance, adherence, and rehabilitation goals. Overall, these interventions were associated with improvements in pelvic floor muscle strength, urinary incontinence and pelvic organ prolapse symptoms, sexual function, psychological status, rehabilitation adherence, and patient satisfaction. Future studies should report intervention dosage more consistently to support clearer comparison and translation into practice.

### 3.3 Multidisciplinary collaboration and team management

Evidence-based nursing in postpartum pelvic floor rehabilitation emphasizes the essential role of multidisciplinary collaboration. The complexity of pelvic floor dysfunction necessitates coordinated involvement from various healthcare professionals, including obstetricians, gynecologists, rehabilitation physicians, physiotherapists, nurses, and psychological counselors (47). Bshawri et al. (11) highlight that the treatment of pelvic floor disorders requires a multidisciplinary approach that integrates expertise from gynecology, nursing, health education, and medical physics, thereby ensuring patient-centered and holistic care.

Within such teams, nurses play a pivotal role as coordinators and implementers. Their responsibilities include conducting assessments, delivering rehabilitation interventions, providing health education, offering psychological support, and facilitating communication among different specialists. Evidence-based care pathways developed in recent studies incorporate principles of team management and structured training for personnel involved in postpartum pelvic floor rehabilitation, reflecting the practical embodiment of multidisciplinary collaboration in evidence-based practice (48). Furthermore, experiential accounts of advanced practice nurses (APNs) working in pelvic floor dysfunction management underscore the importance of multidisciplinary teamwork in enhancing the quality and consistency of evidence-based nursing interventions (12).

### 3.4 Continuous evaluation and quality improvement

Evidence-based nursing is inherently iterative, requiring ongoing evaluation and refinement of care processes. Nurses routinely assess the effectiveness of interventions using outcomes such as pelvic floor muscle strength, symptom reduction, functional improvements, and quality of life measures. For example, indicators including overall rehabilitation effectiveness, pelvic floor muscle grade, and patient satisfaction rates are frequently used to evaluate the impact of postpartum pelvic floor rehabilitation programmes (28). Rehabilitation plans are adjusted promptly based on assessment findings to ensure clinical relevance and intervention efficacy.

**Figure 2:**
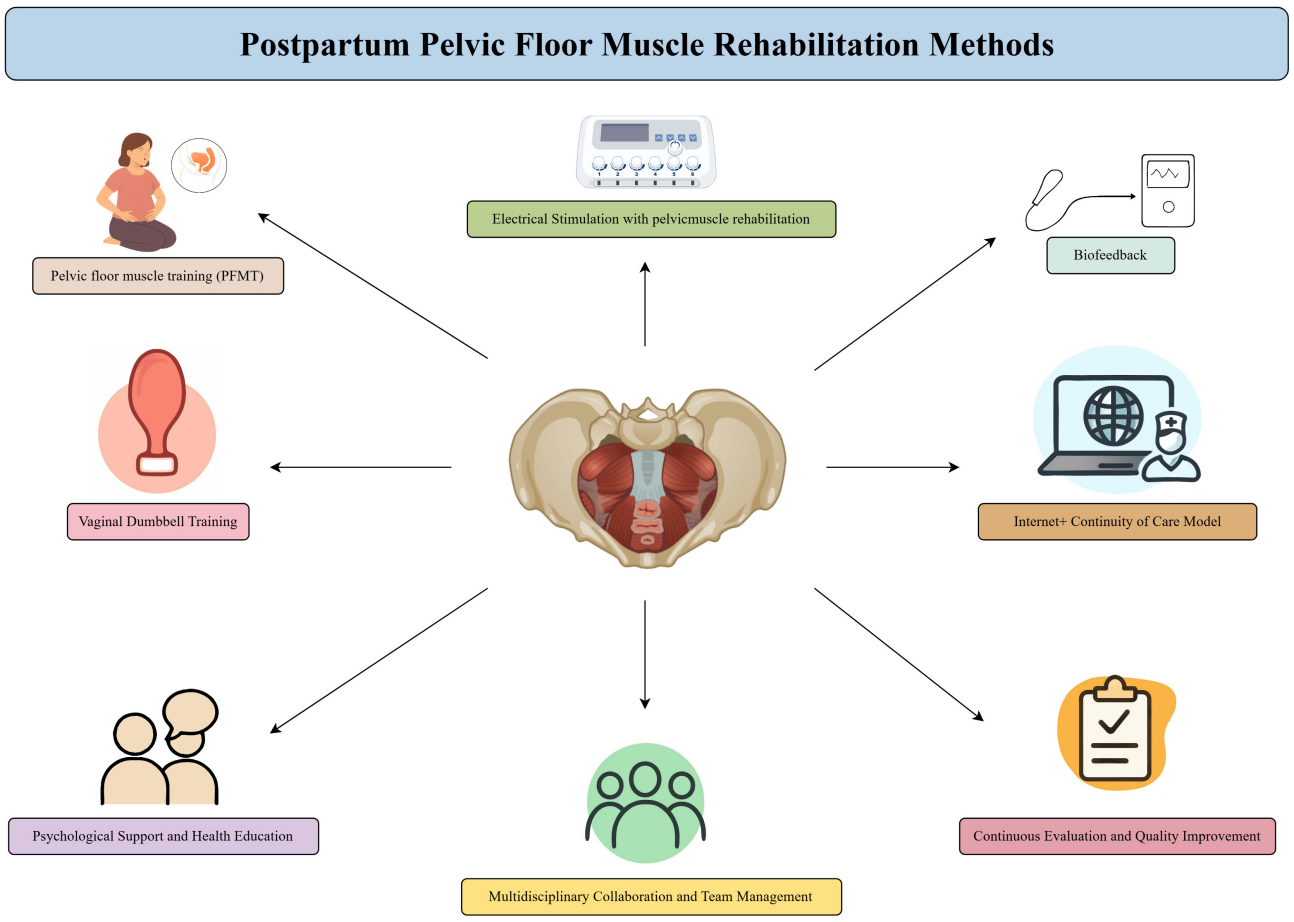
Postpartum pelvic floor muscle rehabilitation methods. PFMT, pelvic floor muscle training; EBN, evidence-based nursing; PFD, pelvic floor dysfunction.

Challenges and insights gained from clinical practice help generate new evidence, driving continuous improvements in postpartum rehabilitation. By combining the best available research with clinical expertise and a focus on patient needs, evidence-based nursing improves care quality, restores pelvic floor function, enhances quality of life, and supports the physical and emotional recovery of postpartum women (see Figure 2).

## 4 Comparative analysis of evidence-based nursing and routine care in postpartum pelvic floor rehabilitation

### 4.1 Comparison of pelvic floor muscle strength improvement

Pelvic floor muscle (PFM) strength is a key indicator of pelvic floor function. Numerous studies have compared evidence-based interventions with routine care and generally suggest that evidence-based nursing approaches may be associated with improvements in PFM strength (as summarized in Table 2).

(1) **Type I and Type II muscle fiber strength:**

**Table 2:** Summary of reported outcome directions for evidence-based and conventional nursing interventions in postpartum pelvic floor rehabilitation.

Intervention	Outcome measures	Evidence-based nursing effect	Conventional nursing effect
Pelvic floor training	Pelvic floor muscle strength	Reported improvement	Modest improvement reported
Biofeedback	Urinary incontinence	Reported improvement	Limited or inconsistent improvement
Electrical stimulation	Sexual function	Reported improvement	Modest improvement reported
Psychological support	Psychological state (anxiety, depression)	Reported improvement	Limited or inconsistent improvement
Health education	Nursing compliance	Reported improvement	Modest improvement reported

Studies report that women receiving low-frequency pelvic floor electrical stimulation combined with biofeedback showed significantly greater improvements in both type I and type II muscle fiber strength at 3 months postpartum compared with those receiving routine care, with markedly higher maximal vaginal pressure (*p* < 0.05) (49). Similar findings indicate that the number of women achieving normal type I and type II contraction strength was significantly higher in the evidence-based intervention group than in the routine-care group (*p* < 0.05) (50), a trend supported by multiple additional studies (51, 52).

**(2) Muscle strength grading and electromyographic values:**

Research involving biofeedback-assisted rehabilitation demonstrates that postpartum women in the intervention group had significantly higher maximal and average electromyographic (EMG) voltages after treatment compared with those receiving routine pelvic floor rehabilitation alone, along with superior PFM strength grades (*p* < 0.05) (53). Huang et al. (54) further found that individualized postpartum rehabilitation guidance produced greater improvements in PFM strength among older postpartum women compared with standard care alone.

### 4.2 Comparison of pelvic floor dysfunction symptom improvement

Evidence-based nursing approaches may offer additional benefits compared with routine care in reducing and preventing symptoms associated with postpartum pelvic floor dysfunction.

**(1) Pelvic organ prolapse and urinary incontinence:**

Multiple controlled studies have shown that evidence-based postpartum pelvic floor rehabilitation significantly reduces the incidence of pelvic organ prolapse, stress urinary incontinence, and pelvic pain compared with routine care (*p* < 0.05), while also improving anatomical measures such as POP-Q staging (19, 31, 55). For instance, one study reported a complication rate of 16.67% in the intervention group vs. 38.33% in the routine-care group (*p* < 0.05) (56). International studies also confirm that pelvic floor rehabilitation effectively alleviates urinary incontinence and related symptoms (57).

(2) **Postpartum urinary retention:**

Studies examining PFMT-based rehabilitation show that the treatment effectiveness rate in intervention groups is significantly higher than in routine-care groups (*p* < 0.05) (27, 58). Additional evidence suggests that integrating supportive psychological interventions with pelvic floor rehabilitation further enhances recovery outcomes for postpartum urinary retention (59).

### 4.3 Comparison of improvements in quality of life and sexual function

Evidence-based nursing approaches have been associated with favorable outcomes in improving postpartum women's quality of life and sexual function.

(1) **Sexual satisfaction and sexual function:**

Multiple studies indicate that evidence-based pelvic floor rehabilitation significantly enhances postpartum sexual function, with higher sexual satisfaction and improved scores on validated instruments such as the Female Sexual Function Index (FSFI), compared with routine care (*p* < 0.05) (28, 60). Additionally, evidence suggests that women receiving evidence-based interventions report lower dyspareunia scores (*p* < 0.05) (61) and show significant improvements in overall quality of life (*p* < 0.05) (54).

(2) **Overall quality of life:**

Interventions grounded in behavioral or theoretical frameworks—such as the Transtheoretical Model and the Information–Motivation–Behavioral Skills model—have been shown to significantly improve postpartum women's multidimensional quality of life. Women in evidence-based intervention groups consistently achieve higher scores on instruments such as the Incontinence Quality of Life Questionnaire (I-QOL) and the GQOLI-74 compared with those receiving routine care (*p* < 0.05) (40, 62–64).

In addition to sexual function and general quality of life, sleep disturbance, social participation, and vocational functioning are also important dimensions of postpartum recovery. Pelvic floor symptoms may interfere with rest, daily activities, interpersonal interaction, and return to work, thereby further reducing women's overall quality of life. However, these outcomes were not consistently assessed in the reviewed studies and therefore could not be analyzed as independent outcome domains in this review.

### 4.4 Comparison of psychological outcomes and treatment adherence

Individualized evidence-based interventions are effective in improving psychological well-being among postpartum women.

(1) **Anxiety and depression:**

Studies show that evidence-based pelvic floor rehabilitation reduces postpartum anxiety and depressive symptoms while enhancing self-efficacy and understanding of the condition (62, 64–66).

(2) **Treatment adherence:**

Interventions informed by behavioral models or innovative care delivery approaches significantly improve adherence to pelvic floor rehabilitation. Qualitative studies further illuminate facilitators and barriers to adherence, offering theoretical guidance for optimizing future interventions (44, 67, 68). Adherence to postpartum pelvic floor rehabilitation is influenced not only by knowledge and access to care, but also by perceived effort, fatigue, childcare demands, time constraints, and motivation. Because many rehabilitation activities require repeated home-based practice, women may find it difficult to maintain regular training even when they understand its importance. Therefore, evidence-based nursing should reduce the perceived burden of rehabilitation by setting individualized goals, providing simple exercise instructions, using reminders and feedback, offering motivational support, and gradually increasing exercise difficulty according to recovery progress.

### 4.5 Safety and timing considerations

Evidence indicates that pelvic floor muscle training, electrical stimulation, and other evidence-based modalities are safe and well tolerated, with no significant adverse effects reported (37, 50). Research further suggests that initiating rehabilitation during the early postpartum period (e.g., 6–13 weeks postpartum) leads to significant improvements in PFM strength and urinary incontinence symptoms, with no meaningful differences observed across varying initiation timeframes (28, 69). Early professional intervention is considered beneficial for both the prevention and management of pelvic floor dysfunction (PFD). However, the optimal timing and duration of combined therapies—such as biofeedback with electrical stimulation—remain inconclusive, highlighting the need for further high-quality research.

### 4.6 Advantages of evidence-based nursing

Evidence-based nursing adopts interventions grounded in the best available research evidence, ensuring scientific rigor and clinical validity. Compared with routine care, evidence-based approaches may contribute to better outcomes in some studies in pelvic floor muscle recovery and quality of life. By emphasizing individualized care plans, comprehensive health education, and psychological support, evidence-based nursing enhances postpartum women's knowledge, adherence, and overall satisfaction (70, 71). It effectively mitigates negative emotional states such as anxiety and depression, facilitates holistic recovery, and promotes ongoing evaluation and quality improvement to ensure timely adjustments in care delivery and optimize health management.

## 5 Summary and future directions

### 5.1 Summary

Evidence-based nursing emphasizes the integration of the best available research evidence, clinical expertise, and patient values. Its application in postpartum pelvic floor rehabilitation spans the entire continuum of care—from assessment and intervention to outcome evaluation. Compared with traditional nursing approaches, evidence-based interventions have generally been associated with improvements in pelvic floor muscle strength, alleviation of pelvic floor dysfunction symptoms, and enhanced recovery outcomes. Psychological support, structured health education, and internet-based continuity-of-care models have also demonstrated substantial benefits. Through comparative analysis, this review highlights the significant advantages of evidence-based translational nursing in postpartum pelvic floor rehabilitation, supporting its broader clinical implementation.

### 5.2 Future directions

Despite notable progress, several limitations persist in evidence-based translational nursing for postpartum pelvic floor rehabilitation. Current research lacks long-term follow-up data, particularly regarding sustained efficacy, recurrence patterns, and the extended impacts of interventions on pelvic floor health among postmenopausal women. Future studies should include prolonged follow-up and larger sample sizes to address these gaps comprehensively. Another limitation is that insomnia, social participation, and vocational functioning were not examined as independent outcome domains in this review, although they may be affected by postpartum pelvic floor symptoms and may contribute substantially to quality of life. Future studies should include these broader quality-of-life indicators to better evaluate the holistic impact of postpartum pelvic floor rehabilitation.

Most existing studies focus on single or relatively narrow intervention components. Moving forward, research should explore more diversified and integrative rehabilitation strategies, such as combining structured exercise programmes (72, 73), physiotherapy modalities including magnetic stimulation (10), and complementary approaches such as traditional Chinese medicine. Moreover, comorbid postpartum conditions, such as lumbopelvic and low back pain, should receive greater attention within rehabilitation protocols, as these symptoms may influence exercise tolerance, adherence, functional recovery, and overall quality of life. Future postpartum pelvic floor rehabilitation programmes should therefore consider pain-related limitations when designing individualized exercise intensity, progression, follow-up, and safety monitoring (74). The development of intelligent, technology-assisted rehabilitation systems also represents a promising direction for enhancing the precision and efficiency of pelvic floor rehabilitation. In future practice, artificial intelligence may further support postpartum pelvic floor rehabilitation by assisting individualized risk stratification, adherence monitoring, adaptive exercise progression, and integration of multimodal information, such as symptoms, pelvic floor muscle function, wearable activity data, and patient-reported outcomes. Evidence from AI-assisted physical activity intervention research supports the potential of AI for personalization, monitoring, and intervention evaluation, although this evidence should be applied cautiously because it is not specific to postpartum pelvic floor rehabilitation (75, 76). Future studies should therefore evaluate the safety, feasibility, interpretability, privacy protection, and clinical effectiveness of AI-supported pelvic floor rehabilitation programmes.

Multidisciplinary collaboration will remain essential in advancing this field. Future research should delineate the specific roles and contributions of each discipline within collaborative care frameworks to strengthen the overall capacity for evidence-based practice among clinical teams. In addition, increased emphasis should be placed on prenatal pelvic floor muscle training and preventive health education to reduce the incidence of postpartum pelvic floor dysfunction.

Overall, evidence-based translational nursing in postpartum pelvic floor rehabilitation has demonstrated substantial clinical value and considerable potential for future development. Continued high-quality research will be vital for establishing comprehensive, effective, and globally applicable pelvic floor health management strategies for women across the lifespan.
